# Mapping of Health Literacy and Social Panic Via Web Search Data During the COVID-19 Public Health Emergency: Infodemiological Study

**DOI:** 10.2196/18831

**Published:** 2020-07-02

**Authors:** Chenjie Xu, Xinyu Zhang, Yaogang Wang

**Affiliations:** 1 School of Public Health Tianjin Medical University Tianjin China

**Keywords:** COVID-19, China, Baidu, infodemiology, web search, internet, public health, emergency, outbreak, infectious disease, pandemic, health literacy

## Abstract

**Background:**

Coronavirus disease (COVID-19) is a type of pneumonia caused by a novel coronavirus that was discovered in 2019. As of May 6, 2020, 84,407 cases and 4643 deaths have been confirmed in China. The Chinese population has expressed great concern since the COVID-19 outbreak. Meanwhile, an average of 1 billion people per day are using the Baidu search engine to find COVID-19–related health information.

**Objective:**

The aim of this paper is to analyze web search data volumes related to COVID-19 in China.

**Methods:**

We conducted an infodemiological study to analyze web search data volumes related to COVID-19. Using Baidu Index data, we assessed the search frequencies of specific search terms in Baidu to describe the impact of COVID-19 on public health, psychology, behaviors, lifestyles, and social policies (from February 11, 2020, to March 17, 2020).

**Results:**

The search frequency related to COVID-19 has increased significantly since February 11th. Our heat maps demonstrate that citizens in Wuhan, Hubei Province, express more concern about COVID-19 than citizens from other cities since the outbreak first occurred in Wuhan. Wuhan citizens frequently searched for content related to “medical help,” “protective materials,” and “pandemic progress.” Web searches for “return to work” and “go back to school” have increased eight-fold compared to the previous month. Searches for content related to “closed community and remote office” have continued to rise, and searches for “remote office demand” have risen by 663% from the previous quarter. Employees who have returned to work have mainly engaged in the following web searches: “return to work and prevention measures,” “return to work guarantee policy,” and “time to return to work.” Provinces with large, educated populations (eg, Henan, Hebei, and Shandong) have been focusing on “online education” whereas medium-sized cities have been paying more attention to “online medical care.”

**Conclusions:**

Our findings suggest that web search data may reflect changes in health literacy, social panic, and prevention and control policies in response to COVID-19.

## Introduction

In December 2019, a severe public health emergency was induced by the outbreak of a novel coronavirus, which has since been named coronavirus disease (COVID-19) by the World Health Organization (WHO) [1]. Since the first-level response by government officials to COVID-19 across China’s provinces and cities, governmentally imposed social isolation has provided the Chinese population with ample time to search online for the latest COVID-19–related news [2]. Baidu, as the most widely used Chinese search engine, accounts for two-thirds of China’s search engine market share [3]. At the time of the COVID-19 outbreak, we found that the number of searches for COVID-19 had increased exponentially, despite the fact that its incidence and mortality rates were much lower than those of some noncommunicable diseases, such as cancer. Hence, this phenomenon is worthy of further attention and discussion.

## Methods

We obtained web search data from the Baidu Index [4]. As of May 2020, Baidu accounts for 71.23% of the search engine market share in China [5] and is the most widely used search engine in the country. It is a well-known and extensive platform for information/resource sharing that Chinese internet users rely on.

Search data, dating to as early as 2004, were derived from search frequencies on Baidu. Frequencies were calculated based on the search volumes of specific search terms entered by internet users, and data on a daily, monthly, and yearly basis were obtained for the search terms we chose from the Baidu Index.

We entered the formal Chinese names for “lung cancer,” “liver cancer,” “esophageal cancer,” “colon and rectum cancer,” and “breast cancer,” respectively, and acquired their search data history from January 1, 2020, to March 17, 2020. The Baidu Index provides search term analysis; it also uses a process to scientifically determine related search terms based on the mode through which the searchers initiate a search request. This means that other relevant search terms will be provided automatically once the internet users enter a search term. Thus, we entered “COVID-19” into the Baidu Index and obtained its related search terms from February 11, 2020, to March 17, 2020 (search volumes for “COVID-19” can only be obtained from February 11, 2020 onward). The search terms mainly included health literacy, social panic, and prevention and control measures relating to COVID-19. All search data were downloaded free of charge on March 17, 2020.

We performed descriptive analyses to describe and compare the overall search situation under the context of the pandemic from different aspects. Statistical analysis was conducted using Excel 2016 (Microsoft Corporation) and IBM SPSS (version 22.0, IBM Corporation). We used Tableau (version 2018.3, Tableau Software) and Excel 2016 (Microsoft Corporation) to create heat maps.

## Results

### Mapping of Health Literacy and Social Panic Via Web Search Data During a Public Health Emergency

The COVID-19 pandemic necessitates requirements for public health literacy. Despite China’s active response in combating this outbreak, a proportion of the Chinese population has remained afraid, which may seriously affect progress in controlling and preventing future outbreaks. This phenomenon is particularly striking on the internet and within social media, and investigating COVID-19–related health literacy and social panic via these platforms may yield important insights.

Taking the COVID-19 outbreak as an example, spurious information disseminated via social media by nonexpert individuals has included false claims on how to kill the COVID-19 virus (severe acute respiratory syndrome coronavirus 2 [SARS-CoV-2]), for example, by drinking alcohol, smoking, aromatherapy, essential balms, use of a hair dryer, or taking a hot bath [2]. The internet has figuratively become a double-edged sword in the context of the COVID-19 outbreak.

### Concerns of Internet Users During the COVID-19 Outbreak

By summarizing web searches for COVID-19, the most searched content has focused on the progression of the pandemic and tips on how to protect oneself from infection [6]. Web searches on COVID-19 have mainly included the following: the latest news on the pandemic; relevant knowledge about COVID-19 infection prevention and control; basic, as well as more comprehensive, information describing SARS-CoV-2; and rumors and suggested policies from unqualified sources [6].

**Figure 1:**
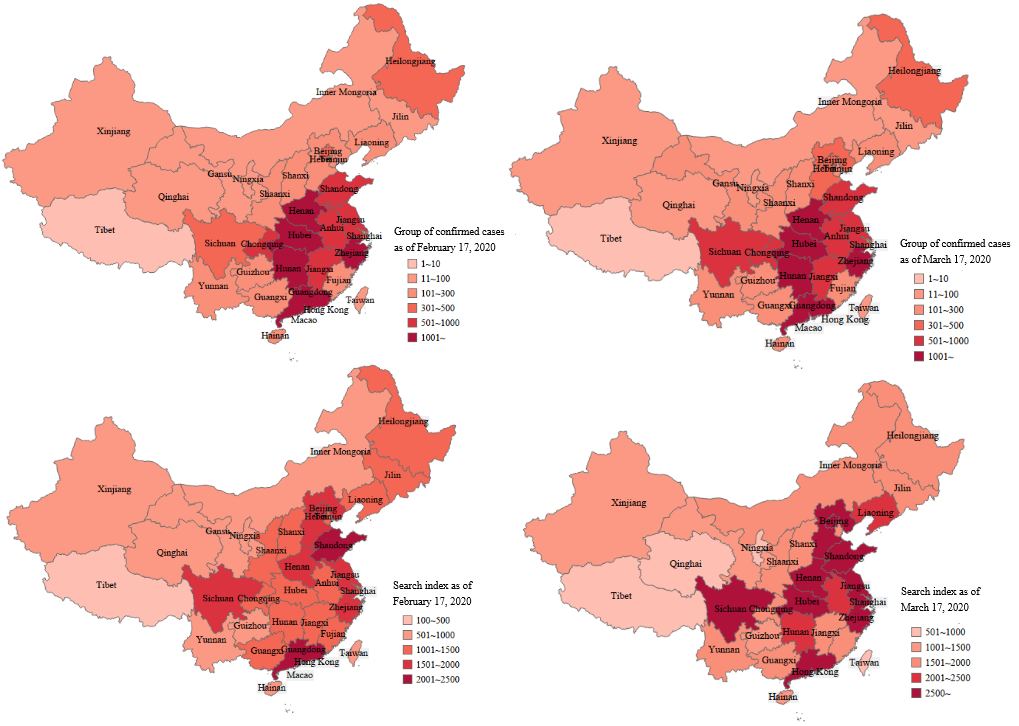
The cumulative number of confirmed cases of coronavirus disease (COVID-19) (top row) and the change in COVID-19's search index (bottom row) in China.

Citizens in Wuhan, Hubei Province, have been more concerned about COVID-19 compared to citizens from other cities during the same period since the outbreak first occurred in Wuhan (Figure 1). Wuhan citizens searched for content related to “medical help” 22% of the time, followed by searches for “protective materials” and “pandemic progress.” Medical help refers to special assistance and support provided to the citizens of Wuhan during the pandemic, which included the deployment of 42,000 trained medical workers from across the country to Wuhan and other cities in Hubei Province, and donations of medical supplies (medical masks, medical protective clothing, ventilators, etc).

Since the COVID-19 outbreak, web searches for wild animals have reached a historical peak—the Baidu topic “refuse to eat wild animals” has reached nearly 100 million views [7]. Content related to “harm to wild animals” has received considerable attention. At the early stage of the pandemic, there were some speculation that wild animals may have spread the coronavirus to people in Wuhan, although there is no scientific conclusion regarding the origin of COVID-19 [8-10]. In this context, on February 24, 2020, on the basis of the *Law of the People's Republic of China on the Protection of Wildlife*, China established a comprehensive system to prohibit the consumption of wild animals [11].

With the pandemic now gradually under control in China, public concerns have changed accordingly [12]. Web searches for “when to return to work and start school” have increased eight-fold from those during the previous month based on the web search data in the Baidu Index. Searches for content related to “closed community and remote office” have continued to rise, and searches for “remote office demand” have risen by 663% from the previous quarter. Individuals from first-tier cities such as Beijing, Shanghai, Shenzhen, Guangzhou, and Chengdu have paid more attention to telecommuting. Employees who have returned to work have mainly engaged in web searches on “return to work and prevention measures,” “return to work guarantee policy,” and “time to return to work.” Men have been half as attentive as women regarding protective measures related to returning to work.

Previously, the four major industries most discussed by Chinese netizens have been online education, online medical care, online entertainment, and fresh electronic commerce (e-commerce) (Table 1) [6]. During the pandemic, provinces with large populations of educated individuals (eg, Henan, Hebei, and Shandong) have been focusing on “online education.” In contrast, fourth-tier cities have been paying more attention to “online medical care” (in China, cities are usually graded according to various levels such as city development, comprehensive economic strength, talent attractiveness, information exchange capability, international competitiveness, technological innovation capability, and transportation accessibility. First-tier cities refer to metropolises that are important for social, political, and economic activities [eg, Shanghai, Beijing]. Most fourth-tier cities are medium-sized cities.). Additionally, young and middle-aged groups exhibited greater search volumes for “online medical care” [10].

**Table 1:** Growth rate of search volumes of the four major industries discussed by netizens [6].

Industry	Search growth rate (%)
Online education	248
Online medical care	200
Online entertainment	170
Fresh electronic commerce	120

### Impact of a Lack of Health Literacy on the COVID-19 Outbreak

COVID-19 is a novel and highly infectious disease [13,14], but in the early days of the outbreak, people had limited knowledge of it and did not know how to prevent it, which concomitantly caused panic. Despite the COVID-19 pandemic becoming gradually controlled in China, most people still remain under high alert. Some misinterpretations of discussions on the resumption of work on the internet have caused another wave of panic among some groups and cities since returning to work. This phenomenon can be observed on Weibo, a social media platform based on information sharing, dissemination, and acquisition in real time (similar to Facebook). Many people are worried about whether a large number of people returning to work will reinitiate the spread of COVID-19. However, individuals in areas with no outbreaks of the disease or low incidence of infections have also expressed this concern. However, if cities continue to stagnate, such stagnation may lead to a higher mortality rate than that attributed to COVID-19. Zhang Wenhong, head of a Shanghai medical treatment expert group, stated the following in an interview [15]:

If the hospital does not return to work, cancer patients cannot receive chemotherapy and surgery, and other infected people cannot be treated. Patients with trauma cannot get a good treatment. Under such circumstances, the number of patients dying from other diseases will far exceed the number of people dying from the new coronavirus.

### Why Are Public Health Emergencies of Greater Concern Than Highly Fatal Chronic Diseases Like Cancer?

Cancer-related deaths in China account for approximately 27% of all global cancer-related deaths [16,17]. As presented in Table 2, except for esophageal cancer, the search frequencies of all other cancers have decreased since the COVID-19 outbreak. Meanwhile, the search frequency of COVID-19 has increased significantly [4]. Dr Tedros Adhanom Ghebreyesus, Director-General of the WHO, reported that the global mortality rate of COVID-19 is approximately about 3.4% [18]. According to the latest data from the National Health Commission of the People’s Republic of China on February 3, 2020, the mortality rate of COVID-19 in Hubei Province is 3.1%, while the national COVID-19 mortality rate is even lower at 0.2% [19].

**Table 2:** Changes in the search index of the top five cancers and coronavirus disease (COVID-19) in China from January 27, 2020, to March 17, 2020.

Disease	Incidence rate (per 100,000 persons)^a^	Mortality rate (per 100,000 persons)^a^	Daily mean value		Overall search index^b^		Mobile search index^b^	
			Overall search index	Mobile search index	Year-on-year change (%)	Month-on-month change (%)	Year-on-year change (%)	Month-on-month change (%)
Lung cancer	58	49	3849	3538	–23	–1	–19	—^c^
Liver cancer	37	30	1301	1128	–63	–22	–65	–18
Esophageal cancer	17	15	1108	957	11	3	14	3
Colon and rectum cancer	31	13	215	120	–12	–9	–21	–16
Breast cancer	26	6	2235	2034	–50	–15	–50	–13
COVID-19	—	—	25,256	19,614	—	4263	—	3784

^a^Incidence and mortality rates for the five cancers were obtained from the Global Burden of Diseases Database [20].

^b^Negative values represent decline.

^c^Not available.

The death rate associated with cancer is much higher than that of COVID-19. If we use the national data (excluding Hubei Province), the mortality rate of COVID-19 is comparable to that of the general influenza, and the total incidence rate is far lower than that of influenza [21,22]. There are several reasons that may explain the relatively high mortality rates of COVID-19 in Wuhan and in Hubei Province in general, including the stronger virulence of SARS-CoV-2 in Wuhan, more cross-infection, and the prevalence of patients with mild symptoms who did not see a doctor [23].

## Discussion

### Principal Findings

Health literacy includes two aspects: (1) knowledge, which comprises basic health knowledge and skills; and (2) ability, which refers to one’s ability to acquire, understand, screen, and apply health information [24,25]. Health information literacy represents the core of health literacy—it can greatly improve the public's capacity for self-protection in order to improve the overall response to public health emergencies.

The health literacy rate of Chinese residents in 2018 was 17.06% [26]. It mainly covers basic health knowledge and concept literacy, healthy lifestyle and behavior literacy, and basic health skills literacy. Although the health literacy rate of residents has improved, the uneven distribution of health literacy levels between urban and rural areas, and across regions and populations, still exists. The health literacy levels of rural residents, residents in the Midwest, and the elderly are relatively low. As mentioned above, with the rapid development of internet technology, people can easily use the internet to search for health information. However, the new coronavirus that caused the recent pandemic was previously unknown. Since the COVID-19 outbreak, the related transmission characteristics, symptoms, transmission channels, and methods for protection have been gradually communicated to the public via recent publications on COVID-19–related research. Public health emergencies have the characteristics of urgency and paroxysm since they require the public to respond quickly. At such times, the ability to acquire, understand, and use health data will enable individuals to more quickly facilitate disease control and prevention in the face of a public health emergency.

People use the internet for almost everything they do nowadays. By uploading and downloading information, everyone can be a publisher and conveyor of the immense quantity of information available on the internet. Search engines facilitate the acquisition and learning of health information from a variety of sources, which provides the public with more diversified content, autonomy, and greater control over choices. However, search engines also lead to many problems. For example, they may disseminate data from unreliable sources, making it difficult for the public to distinguish between high-quality and low-quality health information [27-29]. In these unprecented times, people are more vulnerable and credulous to the impact of new information related to COVID-19. Hence, the vast amount of information available on the internet undoubtedly has a considerable impact on public health literacy and may influence the control and prevention of further outbreaks.

Improving health information literacy is the primary component of improving overall health literacy. First, it is necessary to raise awareness of the important role of health information literacy, and to realize how it can improve public health and promote the development of health services. Second, the state should take the lead in setting up a network containing national health information and support services from multiple sources (eg, the public, medical organizations, governmental sectors) to ensure that the public can receive assistance in obtaining health information and related skills. Because health information involves professional knowledge and also concerns the health of every citizen, the release of authoritative information from professional agencies during the pandemic is particularly important. During the COVID-19 pandemic, many users of social media often cited the words of academician Zhong Nanshan, a trusted public academic, to spread information from a credible source. This pattern demonstrates that individuals need authoritative health information. Timely and authoritative information may be one of the most effective ways to eliminate doubt and reduce panic, especially in the face of public health emergencies,. Third, education should be strengthened to improve the level of public health literacy throughout schools, communities, and villages. Individual education levels and education systems are important factors affecting public health information literacy, and health information literacy education through comprehensive linkage represents the most inclusive and cost-effective precautionary measure [30,31]. As recommended by the WHO, the following response strategies are required: rapidly establishing international coordination and operational support; scaling up of national readiness and responses to operations; and accelerating priority research and innovation [32].

In addition to face-to-face communication, the online role of health care providers in public health communications is also important for mitigating medical misinformation. Examples of such online public health communications are WebMD in the United States, AskDr in Singapore, HaoDF in China, etc. Through these outlets, health care authorities can communicate with the public via the internet and provide professional and reliable health information.

While classic public health measures still play very important roles in tackling the current COVID-19 pandemic, there are also many new potentially enabling technological domains that can be applied to help monitor, survey, detect, and prevent such pandemics, including the Internet of Things, big data analytics, artificial intelligence, and blockchain technology. Therefore, we should make full use of these emerging technologies to contain the current COVID-19 outbreak, as well as future pandemics in a timely and effective manner [33-39].

### Conclusions

Since the Chinese New Year, the COVID-19 outbreak has become the most important issue in China. Fear of an unknown virus represents the beginnings of panic, and the internet and social media networks have since become incubators and catalysts of panic. Individual cases can be shared by tens of millions of people in a single day through social media dissemination. Hundreds of millions of people are eagerly absorbing information about the novel coronavirus, but reading about new cases is undoubtedly causing concerns among citizens about their own health status.

The pandemic has diverted attention from other health issues. China accounts for 19% of the global population, and its incidence of cancer accounts for 22% of the total global prevalence of cancer. Nevertheless, these diseases have never caused panic at the level of COVID-19. The mortality rate of COVID-19 cannot be compared with that of any other major noncommunicable disease. Despite this discrepancy, the Chinese population continues to be horrified by COVID-19 but at ease with other known diseases. We need to collectively alter our minds about COVID-19 in order to manage such public health emergencies more rationally. This change requires a more logical use of information obtained from social media and the internet during public health emergencies, as well as improvements in health literacy and the ability to cope with social panic.

## Abbreviations

COVID-19: coronavirus disease
e-commerce: electronic commerce
SARS-CoV-2: severe acute respiratory syndrome coronavirus 2
WHO: World Health Organization
